# Cognitive Processing Speed, Working Memory, and the Intelligibility of Hearing Aid-Processed Speech in Persons with Hearing Impairment

**DOI:** 10.3389/fpsyg.2017.01308

**Published:** 2017-08-15

**Authors:** Wycliffe Kabaywe Yumba

**Affiliations:** ^1^Department of Behavioral Sciences and Learning, Linköping University Linköping, Sweden; ^2^Linnaeus Centre HEAD, Swedish Institute for Disability Research, Linköping University Linköping, Sweden

**Keywords:** aging, cognition, speech recognition in noise, hearing aid, signal processing algorithms, hearing impairment

## Abstract

Previous studies have demonstrated that successful listening with advanced signal processing in digital hearing aids is associated with individual cognitive capacity, particularly working memory capacity (WMC). This study aimed to examine the relationship between cognitive abilities (cognitive processing speed and WMC) and individual listeners’ responses to digital signal processing settings in adverse listening conditions. A total of 194 native Swedish speakers (83 women and 111 men), aged 33–80 years (mean = 60.75 years, *SD* = 8.89), with bilateral, symmetrical mild to moderate sensorineural hearing loss who had completed a lexical decision speed test (measuring cognitive processing speed) and semantic word-pair span test (SWPST, capturing WMC) participated in this study. The Hagerman test (capturing speech recognition in noise) was conducted using an experimental hearing aid with three digital signal processing settings: (1) linear amplification without noise reduction (NoP), (2) linear amplification with noise reduction (NR), and (3) non-linear amplification without NR (“fast-acting compression”). The results showed that cognitive processing speed was a better predictor of speech intelligibility in noise, regardless of the types of signal processing algorithms used. That is, there was a stronger association between cognitive processing speed and NR outcomes and fast-acting compression outcomes (in steady state noise). We observed a weaker relationship between working memory and NR, but WMC did not relate to fast-acting compression. WMC was a relatively weaker predictor of speech intelligibility in noise. These findings might have been different if the participants had been provided with training and or allowed to acclimatize to binary masking noise reduction or fast-acting compression.

## Introduction

Hearing-impaired individuals often show increased difficulties recognizing speech under adverse listening conditions, including noise and reverberant or distorted speech, even when wearing hearing aids (Committee on Hearing, Bioacoustics, and Biomechanics [CHABA], 1988; Akeroyd, 2008; Larsby et al., 2008; Souza and Arehart, 2015). Previous studies have indicated that older adult hearing aid users may have difficulties handling the consequences of hearing aid signal processing, whether in regard to the distortions caused by the effects of background noise or the unwanted artifacts from certain digital signal processing algorithms (e.g., fast-acting compression; Lunner, 2003; Souza and Arehart, 2015). These consequences may lead to benefits of signal processing in the hearing aid that are less than expected. It may be possible that the signal processing implemented in the hearing aid itself may be cognitively demanding for hearing-impaired hearing aid wearers (Lunner, 2003). Others studies have attributed speech recognition difficulties among hearing-impaired hearing aid users to the slow-down of cognitive speed. This decline in cognitive processing speed may arise from a generalized slowing in brain functioning due to advancement in age, which could be responsible for most, if not all, age-related declines in problem-solving, memory, and language comprehension (Salthouse, 1996; Pichora-Fuller, 2003; Schneider et al., 2005).

Working memory refers to a cognitive system responsible for processing and temporary storage of information during complex cognitive tasks, such as comprehension, learning and reasoning (Baddeley and Hitch, 1974; Daneman and Carpenter, 1980; Baddeley, 1986). This memory system is assumed to have a limited capacity that need be shared between the work and the memory, between the processing and storage demands of the task to which the working memory is applied (Daneman and Carpenter, 1980; Baddeley, 2012). Working memory capacity (WMC) is generally assessed with span tests, such as reading span test (Daneman and Carpenter, 1980), semantic word-pair span test (Rönnberg et al., 2016). Cognitive processing speed (CPS) is the rate at which relatively simple perceptual and automatic cognitive operations can be carried out; usually this is measured under time pressure in such that a degree of focused attention is involved (Salthouse, 1996, 2000). Several types of tasks are used to measure cognitive processing speed ability, including lexical decision speed test (LDT), rapid automatized naming test (RAN), physical matching test (PMT), Rhyme judgment test, the digital symbol substitution test (DSST), the flanker task, etc. (Wingfield et al., 1985; Rönnberg, 1990; Salthouse, 1996; Wiig et al., 2002). For example, LDT is considered as one of the most often used tasks in the field of visual word recognition. In this task, participants have to judge as quickly and accurately as possible whether a displayed letter string is a real word or a non-word (Rönnberg, 1990). The overall assumption that underlies the use of LDT is that the rate and the accuracy of reacting to word stimuli shows the effectiveness with which word representations are activated or retrieved from long-term lexical memory (Rönnberg, 1990). Previous studies suggested that cognitive processing speed may be affected by individuals’ knowledge base and experience. That is, the more an individual knows about something and/ or the experience she/he has with it, the greater the probability that her/his cognitive processing speed on tasks related to this information will be increased (Lunner, 2003; Pichora-Fuller, 2003; Desjardins and Doherty, 2014).

A comprehensive way of viewing the relationship between working memory and speech recognition is via the Ease of Language Understanding (ELU) model (Rönnberg et al., 2008, 2013). The ELU model considers language input as consisting of phonological, syntactic prosodic, and semantic information. That is, when speech signal input is degraded or altered from its ordinary form, it can be more difficult to match those acoustic patterns to phonological representations stored in the long term memory, and working memory may be explicitly engaged to a greater extent to reconcile a match (mismatch, Rönnberg et al., 2008). However, under favorable conditions, the incoming speech signal input are not degraded (audible or undistorted), it can be easily matched to a phonological representation stored in long -term memory); and WMC may be engaged to a lesser extent (Rönnberg et al., 2013). In the context of this model, signal degradation refers to whatever may substantially modify the available acoustic signal cues of the target signal (Rönnberg et al., 2008). The sources of signal degradation may be single (e.g., noise) or multiple (e.g., combined hearing aid signal processing and noise for older persons with hearing loss). Other studies suggest that for listeners with hearing loss, various signal processing algorithms implemented in hearing aids may be a potential source of speech signal degradations (Gatehouse et al., 2003, 2006; Foo et al., 2007; Souza and Arehart, 2015).

Modern digital hearing aids are typically equipped with a wide range of signal processing algorithms, including wide dynamic range compression speed, noise reduction, and directional microphones (Dillon, 1996, 2001, 2012; Kates, 2008). Although many hearing-impaired persons may benefit from such signal processing algorithms, they may introduce distortions that may counteract or reduce the intended benefits for some listeners (Lunner, 2003). Fast-acting wide dynamic range compression (fast-acting WDRC) is intended to simultaneously improve the audibility of weak sounds and maintain loudness and comfort for higher-intensity sounds (Dillon, 2001, 2012). Moreover, improved audibility requires signal modification, and a greater modification of the expected acoustic signal may place greater demand on WMC (Rönnberg et al., 2008, 2013). Fast-acting compression may modify the speech amplitude envelope, which may cause a challenging listening situation for hearing-impaired persons who rely on envelope cues (Kates, 2008; Dillon, 2012). In addition, other studies suggest that fast-acting compression may introduce unwanted artifacts, which may create greater signal modification (Dillon, 2001; Lunner, 2003). A number of studies found a relationship between cognitive abilities and the ability to recognize speech in noise using different types of hearing aid signal processing algorithms. In particular, these studies showed that WMC was associated with speech recognition in noise performance when spoken sentences were amplified by fast-acting wide dynamic range compression (Gatehouse et al., 2003, 2006; Foo et al., 2007; Lunner and Sundewall-Thorén, 2007; Akeroyd, 2008; Ohlenforst et al., 2015; Souza and Arehart, 2015). Other studies indicated that WMC, executive function and cognitive speed were related to wide dynamic range compression (Schwartz et al., 2008; Souza and Arehart, 2015) and to frequency compression (Arhart et al., 2013; Souza and Arehart, 2015). Rudner et al. (2011) found that hearing impaired listeners with lower WMC demonstrated poor benefit with fast-acting compression than slow-acting compression, compared with listeners with higher working memory who benefited more with fast-acting compression (see also Foo et al., 2007). A study by Gatehouse et al. (2003) indicated that cognitive capacity was associated with speech recognition in noise with fast-acting compression. That is, there was a greater benefit from fast-acting compression for listeners with greater cognitive capacity than those with poorer cognitive ability in modulated noise background. In further study, the same authors (Gatehouse et al., 2006) reported that cognitive capacity related to speech recognition in noise performance differently (both with fast and slow acting compression), suggesting that fast-acting compression provided greater benefit for listeners with larger cognitive capacity, while slow-acting provided better benefit for listeners with smaller cognitive capacity.

The rationale for noise reduction algorithms is to identify and suppress the adverse effects of background noise on speech recognition and sound quality by improving the signal-to-noise ratios (SNRs) for listeners with hearing loss (Kates, 2008; Dillon, 2012). Although, noise reduction systems are intended to improve speech intelligibility, they may also affect speech quality and ease of listening by introducing signal distortions (Kates, 2008; Wang et al., 2009; Souza and Arehart, 2015). A few studies found a relationship between WMC and spoken sentences amplified by digital noise reduction (Ng et al., 2013, 2015; Arhart et al., 2015). Recently, Ng et al. (2013) conducted a study where they examined the effects of WMC, noise and binary mask-based noise reduction on speech recognition and recall. They found that listeners with larger WMC were better at recalling more words than listeners with smaller WMC, as a result of noise reduction processing. The results showed that noise reduction effectively suppressed the adverse effects of background noise on speech recall performance of listeners with larger WMC. In another recent study, Ng et al. (2015) carried out a research where they tested the Non-ideal version of noise reduction in a follow-up experimental based on essentially the same set-up with elderly hearing aid users. Arhart et al. (2015) investigated the effects of ideal binary mask-based noise reduction processing and several non-ideal versions resulting from the systematic manipulations of two algorithmic parameters. The results showed that WMC was a potential predictor of the overall speech intelligibility performance; however, there was no interaction between WMC and the level of signal distortions in explaining the performance. Related to WMC, recent study by Neher (2014) examined the effects of WMC and hearing loss on response to noise reduction for the three levels of a binaural coherence algorithms (i.e., none, moderate, and strong). They found that speech recognition performance was poor when speech was amplified with noise reduction, and there was no significant difference between listeners with larger WMC and those with smaller WMC. Nevertheless, working memory appeared to be important for the fact that participants with smaller WMC preferred more aggressive (strong) noise reduction than moderate noise reduction (in terms of speech quality).

A number of studies suggested that the association between working memory and noise reduction is likely to be stronger for speech recognition performance under low-context speech material in modulated background noise and relatively weaker under unmodulated background noise (Rudner et al., 2011; Ng et al., 2013). A few studies supporting this view suggested that sentence material may play an important role. For example, shorter speech segments in relatively favorable signal -to- noise ratios may reduce the activation for engagement for WMC compared with longer speech segments which activate the deployment of working memory to a greater extent (Rudner et al., 2009; Souza and Arehart, 2015). Previous studies have suggested that processing speed, WMC and selective attention are essential for linguistic analysis for speech in challenging listening situations (Lunner, 2003; Rönnberg et al., 2013).

The present study investigated the relationship between cognitive abilities (cognitive processing speed, WMC) and individual listeners’ responses to digital signal processing settings in noise. Here, we manipulated hearing aid signal processing and background noise, resulting in six conditions (see **Table 1**) in which Hagerman sentences were presented. We hypothesized that cognitive abilities would be correlated with Hagerman sentence intelligibility in noise. We would expect stronger associations between WMC and speech recognition when speech is acoustically degraded and weaker associations when speech is audible. Numerous studies have supported this view, showing stronger relationship between WMC and speech comprehension in adverse listening conditions in hearing-impaired participants (Foo et al., 2007; Akeroyd, 2008; Rudner et al., 2011; Rönnberg et al., 2013; Ohlenforst et al., 2015; Souza and Arehart, 2015).

**Table 1:** Means (*M*) and Standard Deviations (*SD*) for speech recognition in noise, age, and cognitive measures.

	*M*	*SD*
Age (years)	60.75	8.89
Lexical decision speed test (LDT, reaction time, in ms)	979.02	202.18
Semantic word-pair Span test (SWPST, max score = 42)	17.47	5.36
**Outcome variables**		
**Hagerman test (dB SNR)**		
Linear amplification, steady state noise (SSN), no noise reduction	-4.08	2.04
Linear amplification, four-talker babble (4TB), no noise reduction	1.42	2.06
Linear amplification, SSN, noise reduction	-8.47	2.19
Linear amplification, 4TB, noise reduction	-5.15	2.02
Non-linear amplification (Fast-acting compression), SSN, no noise reduction	-3.21	2.40
Non-linear amplification (Fast-acting compression), 4TB, no noise reduction	2.27	2.09

## Materials and Methods

### Participants

A total of 194 native Swedish speakers (83 women and 111 men), aged 33–80 years (mean = 60.75 years, *SD* = 8.89), with bilateral, symmetrical mild to moderate sensorineural hearing loss who had completed a LDT and semantic word-pair span test, and the Hagerman matrix sentence test (Hagerman and Kinnefors, 1995) were included in this study. The pure-tone average hearing threshold for both ears at frequencies 0.5, 1, 2, and 4 kHz (PTA4) was 39.23 dB HL (*SD* = 19.64). The participants were randomly selected from the hearing clinic patient registry at the University Hospital of Linköping, where the testing took place, and were invited to participate by letter. Regarding the inclusion criteria, all participants were bilaterally fitted with digital hearing aids with common features such as WDRC, noise reduction, and directional microphones. All participants had used the hearing aids for a minimum of 1 year at the time of testing. The participants were healthy native Swedish speakers, with normal vision or corrected-to-normal vision (wearers of glasses). The participants had no history of otological problems or psychological disorders. The study was approved by the Linköping regional ethics committee (Dnr: 55-09 T122-09). All participants gave written informed consent to participate.

### Cognitive Tests

#### Semantic Word Pair Span Test

A semantic word-pair span task (Rönnberg et al., 2016) is a visual working memory test that does not compared to reading span test include any syntactic elements in the processing and storage components. The test material consists of a series of word-pairs (such *as “Bun, Hippo”*). The list length varied from 2 to 5, with three trials per length. The task was to comprehend word and to recall either the first or the final words in the displayed series of pair words (Baddeley et al., 1985). The word-pairs were displayed on a computer screen at a speed of 800 ms per word. Half of the word-pairs were living thing (e.g., *“cat”*), and half of the word-pairs were no living thing (e.g., “*paper*”). The participants were instructed to read and identify the words representing the living thing on the screen, then press the button stating in which position the word representing the living thing was presented (e.g., *Left–Right*). To press the left button (*green*) if the living thing is at the left side, and press the right button (*red*) if the living thing is at the right side. After each sequence of word-pair, the participants were asked to repeat orally and loudly all the words that were recently presented either at left or right, and should be in the correct order of presentation. The participants’ responses were scored by the experimenter in terms of total number of words correctly recalled. The maximum total score is 42 points.

#### Lexical Decision Speed Test

The LDT (Rönnberg, 1990; Rönnberg et al., 2016) was used to measure the participants’ cognitive processing speed. In the LDT, eighty words presented visually one item at a time on the computer screen were used as test material, 40 of which were real Swedish words and 40 were not. In this task, participants have to decide as quickly and accurately as possible whether visually displayed combinations of letters are real words or not, by pressing the button “yes” for real word or “no” for no word/ pseudo word. For example, when the word (e.g., “SNÖ, snow”) was displayed, the participant pressed “yes,” this is a real Swedish word, but when the letters (e.g., “NÄKK”) was displayed, the participant pressed “NO” this is not a real Swedish word. The response time was set at 5 s, and the word disappeared when the participants pressed the button. Accuracy and speed of performance were measured based on the reaction times for the correct trials.

#### The Hagerman Test

Speech recognition was measured using the Hagerman matrix sentence test (Hagerman and Kinnefors, 1995). Three lists of 10 sentences, highly constrained in their nature, with low semantic redundancy, were used as test material. The sentences all consisted of five Swedish words, and had the following structure: proper noun, verb, number, adjective, and object, in that order. The sentences were presented in two types of background noise, steady state noise (SSN) or four-talker babble (4TB). An experimental hearing aid with three different signal processing features, including three signal processing features (1) linear amplification without noise reduction (NoP, baseline), (2) linear amplification with noise reduction (NR) and (3) non-linear amplification with fast-acting compression (Fast, without noise reduction), fitted based on each participant’s audiogram was employed. 4TB consisted of recordings of two male and two female native Swedish speakers reading different paragraphs of a newspaper text and SSN (i.e., stationary speech-shaped noise with the same long-term average spectrum as the speech material) were used. These two types of background noise were presented at equal root mean square (RMS) levels (Larsby et al., 2005).

### Background Noise

Two types of background noises were used in this study: SSN and 4TB. SSN is the stationary noise speech-shaped noise (i.e., similar long-term average spectrum as HINT sentences, Hällgren et al., 2006). The 4TB is a type of competing speech, consisting of recordings of two males and two females’ native Swedish speakers reading different paragraphs of a newspaper text (Hagerman and Kinnefors, 1995). The speech babble was introduced 3 s before the onset of sentence stimuli and ended 1 s after sentence offset.

### Signal Processing Algorithms Setting

#### Noise Reduction

The primary goal of binary masking noise reduction systems is to counteract the effects of noise on speech recognition and sound quality by improving the SNR for hearing aid users (Wang et al., 2009; Dillon, 2012; Souza and Arehart, 2015). The time-frequency units were recorded using a 64-channel gammatone filter bank and time-windowing. The idea here is that for each time-frequency unit (in binary matrix), there is a decrease of 10 dB; the local SNR of each given time-frequency unit is less than 0 dB, which means that the signal energy is greater than the noise energy. In this way, there is an optimization of the SNR benefits provided by binary masking (Li and Wang, 2009). The present study used a binary masking noise reduction algorithm as a processing condition (Boldt et al., 2008), rather than a non-ideal estimation of noise reduction.

#### Linear Amplification and Compression

Audibility was provided by setting up the hearing aid in such a way that linear amplification was based on the hearing thresholds of each subject, as a function of voice aligned compression. The settings were then modified using software programmed for a linear 1:1 compression ratio corresponding to pure-tone input levels ranging from 30 to 90 dB SPL. Subsequently, all signals and noises were distributed in such a way that the noise level corresponded to the region of the linear compression ratio, ensuring that there was no effect of any compression knee point or output limiting. The primary goal of voice aligned compression, known as curvilinear WDRC, is to reduce compression at a high input level, and to increase compression at low input levels, by using lower compression knee points (ranging from 30 to 40 dB SPL, depending on the frequency region affected and the degree of hearing loss). The loudness data of Buus and Florentine (2001) has contributed in part to this compression model, which focuses on providing better sound quality, while maintaining speech intelligibility, rather than focusing solely on loudness. The attack time of 10 ms and release time of 40 ms (with a compression ratio of 2:1 in all channels) were employed in fast-acting multichannel WDRC conditions.

### Procedure

The data in this study were collected as part of a larger investigation (Rönnberg et al., 2016), which involved three sessions of approximately 3 h each. Data for this study were collected during the first session (background data and pure-tone average hearing threshold data) and the third session (Hagerman test). All testing was administered individually during a 6-week period. Vision correction was used when necessary.

The Hagerman sentences test took place in a sound-treated test booth, and the participants sat on a chair at a distance of 1 m from a single loudspeaker. The master hearing aid was implemented in an anechoic box (Brüël and Kjaer, type 4232), containing an experimental well checked hearing aid. This experimental hearing aid was fitted based on each participant’s audiogram. This enabled audibility, and control of target signal processing settings such as: linear amplification (without noise reduction, and with noise reduction (Wang et al., 2009; Ng et al., 2013) and fast-acting compression (Dillon, 2001, 2012). Two types of background noise were presented at equal RMS levels; these were the modulated speech-shaped noise based on the modulated pattern of 4TB (consisting of recordings of two male and two female native speakers of Swedish reading different paragraphs of a newspaper text) and the steady state speech-shaped noise.

These hearing aid features and the background noise were manipulated to examine the predictions of aided speech recognition in noise in which cognition abilities are challenged. Linear amplification without binary noise reductions (NoP) served as a baseline to clarify the difference between linear amplification with binary NR and non-linear fast-acting compression (with NR) in terms of benefits.

After calibrating the setup, for each participant, a baseline measure was performed using linear amplification without binary masking noise reduction prior to applying linear amplification with binary masking noise reduction and the non-linear amplification fast-acting compression (without noise reduction) setting. The testing began with two lists of 10 sentences used as practice before the test session. Each practice sentence was presented one at a time in a randomized order at a constant 65 dB SPL in two background noise conditions. The order in which the conditions were tested was fully randomized across participants and between tests. Each participant was tested individually, and for each test and participant, an initial SNR of 0 dB was selected to facilitate the familiarization period with a somewhat easy recognition task. In the experimental session, Hagerman sentences were presented as described in the practice session. Three lists of 10 sentences each were presented to each participant in a randomized order for each condition. For each sentence, the participant was asked to repeat as many of the words as possible. The number of words correctly repeated was recorded on a computer terminal. On the basis of word scores, the SNR was automatically adapted using a standard algorithm that applies an interleaved technique to determine individual SNRs for 50 and 80% correct levels of performance, respectively (Brand, 2000). The 50% threshold represents 2.5 words correct out of five, and 4 words correct out of five corresponds to 80% threshold. The randomization process was beneficial because it reduces the possibility of memorizing or guessing the sentence and reduces the overall learning effect and thus increases the degree of reliability of the Hagerman test. Although the speech signal was fixed, the noise level was adaptively adjusted to match the appropriate SNR.

### Data Analysis

Data analysis was conducted using a 3 × 2 within-group analysis of variance design, with digital signal processing algorithm setting (no processing, noise reduction, and fast-acting compression) and noise type (SSN and 4TB) as independent variables, and speech recognition in noise performance as the dependent variable. The relationship between the measures of cognitive speed, WMC and speech recognition in noise performance was analyzed using Pearson correlations. Given that a large number of correlations were computed, the Bonferroni correction was applied in order to control the chance of committing a Type I errors which could increase. To obtain the Bonferroni corrected/adjusted *p*-value, the original α-value [critical value of *p*(0.05) was divided by the number of comparisons on the dependent variable (i.e., 36]. This yields a new *p*-value (0.0014) that controls for family-wise Type I error rate.

A series of hierarchical multiple linear regression analyses were conducted in aided six conditions, respectively, performance on Hagerman sentences test, under the following test conditions: (1) Linear amplification without noise reduction (NoP) in an unmodulated noise background; (2) Linear amplification combined with noise reduction (NR) in an unmodulated noise background; (3) Linear amplification without noise reduction (NoP) in a multi-talker background; (4) Linear amplification combined with noise reduction (NR) in a multi-talker background; (5) Fast-acting compression (Fast) signal processing in an unmodulated noise background (no noise reduction); (6) Fast-acting compression (Fast) signal processing in a multi-talker background (no noise reduction, Rönnberg et al., 2016), to examine the extent to which cognitive speed and WMC may relate to aided speech recognition performance in noise. All the significance levels were set at *p* < 0.05, and *P* < 0.01(two-tailed). All analyses were performed using SPSS statistical package 23.0 for windows.

## Results

Means and standard deviations for the predictor (cognitive speed and working memory) and the mean SNR (dB) for speech recognition in noise in the various conditions are shown in **Table 1**. Lower SNR scores means better speech recognition performance because low SNR shows that the participants correctly identified the speech signal despite a high level of background noise, while high SNR scores indicate that the sentences could only be correctly repeated at low noise levels (Hagerman and Kinnefors, 1995). The scoring method for the Hagerman sentences boosted a level of performance where 80% (i.e., four out of five) of the words in any particular sentence were recognized correctly. That is, optimal performance can be found even if one word in each sentence is meaningless due to under-amplification or masking. Nevertheless, the SNR at which 50% of words correctly recognized (or 2.5 words out of 5 words) was applied for the calculation of the thresholds in accordance with Plomp and Mimpen (1979).

### Speech Recognition in Noise Performance

A two-way, within-participant analysis of variance, which included the digital signal processing algorithm (no processing, noise reduction, and fast-acting compression) and noise type (SSN and 4TB), was conducted. The results revealed a main effect of the digital signal processing algorithm, *F*(2,386) = 2137.82, *p* < 0.001, $\eta_{p}^{2}$ = 0.91, in which the mean SNR for the noise reduction condition (-6.81 dB, *SE* = 0.13) was lower than that for the no processing condition (-1.33 dB, *SE* = 0.13) and the fast-acting compression condition (-0.47 dB SNR, *SE* = 0.14). *Post hoc t*-tests (Bonferroni adjusted for multiple comparisons) showed that the test performance in the linear amplification with noise reduction condition was better (i.e., with a lower average SNR) than that in the linear amplification without noise reduction condition (*p* < 0.001) (Wang et al., 2009; Ng et al., 2013). In addition, performance in the linear amplification with noise reduction condition was better than that in the non-linear amplification with fast-acting compression condition (*p* < 0.001). This suggests that linear amplification resulted in a better speech recognition performance than non-linear amplification with fast-acting compression. There was also a significant main effect of the noise type, *F*(1,193) = 3637.09, *p* < 0.001, $\eta_{p}^{2}$ = 0.95, in which the mean SNR in the SSN condition (-5.25 dB SNR, *SE* = 0.13) was lower than that in the 4TB condition (-0.48 dB SNR, *SE* = 0.12). This indicates that competing speech noise has a stronger masking effect than stationary noise.

**FIGURE 1:**
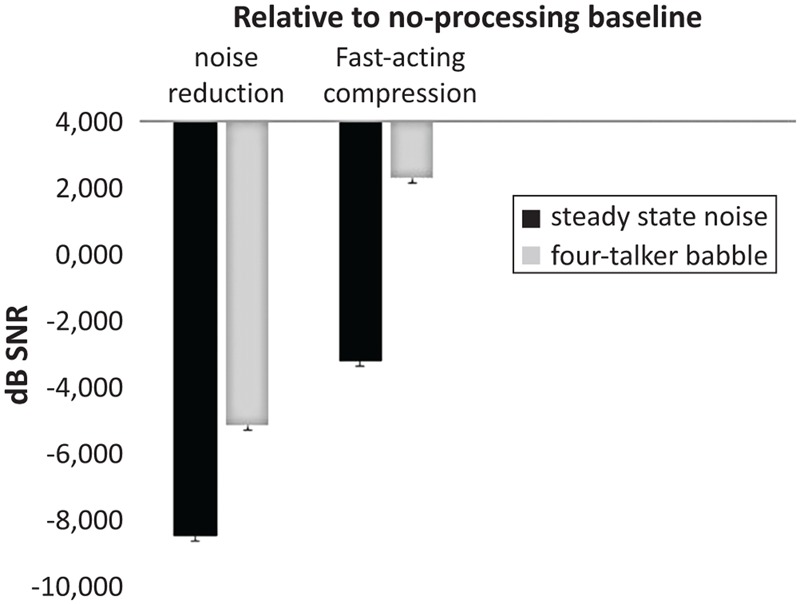
Significant two-way interaction between hearing aid signal processing setting (noise reduction and fast-acting compression) and noise type [steady state noise (SSN), four-talker babble (4TB)] in aided conditions with Hagerman test (relative to NoP baseline, error bars represent standard errors).

**FIGURE 2:**
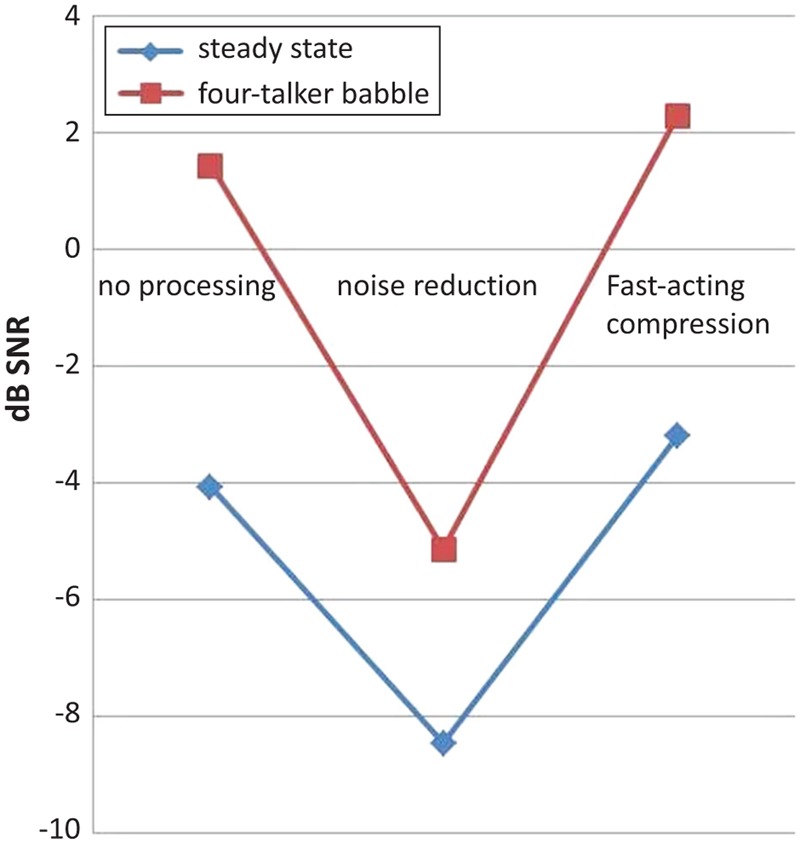
A two-way interaction effect between hearing aid signal processing setting (linear amplification without noise reduction, linear amplification with noise reduction and fast-acting compression) and noise type (SSN, 4TB) in aided conditions with Hagerman test are shown. There was a significant difference between SSN and 4TB (i.e., in dB, in terms of speech recognition performance) in linear amplification without noise reduction, and in non-linear amplification fast-acting compression condition. However, there was no significant difference between SSN and 4TB in linear amplification with noise reduction condition.

Interestingly, a significant two-way interaction effect between the digital signal processing algorithm and the noise type was found, *F*(2,386) = 122.00, *p* < 0.001, $\eta_{p}^{2}$ = 0.38 (**Figure 1**). Further investigation of the interaction using *post hoc t*-testing with Bonferroni adjustment for multiple comparisons showed that the difference between speech recognition performance in SSN and in 4TB was relatively not significant (*p* > 0.05) when binary masking noise reduction was applied. That is, when NR was applied, the presence of noise effect was no longer significant, possibly due to the effectiveness of NR at reducing the masking effect of noise (Ng et al., 2013), compared to when fast-acting compression was applied, where the difference between SSN and 4TB was significant (*p* < 0.05, relative to NoP baseline). As observed in **Figures 1, 2**, this interaction has been driven by the fact that the background noise has a larger effect in the no processing condition (difference of -5.5) and the fast-acting compression (difference -5.48) compared to the in the noise reduction condition (difference of -3.32). This may suggest that background noise rather than cognitive ability is the key factor influencing the interaction (Larsby et al., 2005). We may suggest that there was a relatively smaller dependence on cognitive abilities in the noise reduction condition and a relatively larger dependence on cognitive abilities in fast-acting compression due to the detrimental masking effects of the 4TB condition (e.g., Ng et al., 2013).

The negative masking effects of 4TB might have disrupted and delayed the amplification effectiveness of fast-acting compression (e.g., the compressor speed: attack time or release time) compared to noise reduction. This study indicates that performance was better when spoken sentences were presented in SSN with a noise reduction algorithm. Noise reduction appears to be effective in reducing stable background noise. This is in line with recent studies that suggest that noise reduction provides greater benefits in SSN conditions (Ng et al., 2013; Arhart et al., 2015).

### Correlations

Pearson’s correlations coefficient was calculated to examine the relationship between age, scores on the LDT and the SWPST, and performance on the Hagerman sentences test for the six conditions. Age significantly correlated with the LDT scores (*r* = 0.19, *p* < 0.05) and the SWPST scores (*r* = -0.30, *p* < 0.01). The correlations between age and the cognitive tests suggest that as people become older, cognitive performance becomes worse (e.g., Salthouse, 1996). That is, advanced age corresponds to longer reaction times and lower WMC (Rönnberg et al., 1989; Rönnberg, 1990; Larsby et al., 2005). Age also significantly correlated with the Hagerman sentences test performance under all six aided conditions. This is in line previous studies (e.g., Larsby et al., 2005). The LDT scores also significantly correlated with the Hagerman sentences test performance under all conditions when NoP, NR, and fast-acting compression were used. This finding indicates that cognitive processing speed may be relatively associated with both noise reduction and fast-acting acting compression in terms of speech recognition in noise performance. This pattern of correlations demonstrates the importance of cognitive speed in the ability of hearing aid users to recognize speech in noise.

The performance on the semantic word-pair span scores negatively and significantly correlated with the Hagerman sentences test performance under only 5 out of 6 aided conditions, that is, when NoP, NR, and fast-acting compression were used. This is in line with previous studies that suggested that WMC was related to speech recognition in noise performance with NR (Ng et al., 2013; Arhart et al., 2015) and fast-acting compression (Lunner and Sundewall-Thorén, 2007; Rudner et al., 2011). However, the fact that the semantic word-pair span did not significantly correlate with the Hagerman sentences test performance when there was no processing and in SSN with fast-acting compression contrasts with the findings of Gatehouse et al. (2006) and Lunner and Sundewall-Thorén (2007; see also Ng et al., 2013). The lexical decision speed and semantic word-pair span significantly correlated, showing that a longer reaction time on speech recognition performance is associated with lower WMC.

### Results after Bonferroni Correction Was Applied

When Bonferroni correction was applied, a new *p*-value was obtained (0.0014), and to determine whether any of the corrections was significant, the *p*-value must be *p* ≤ 0.001. The correlations between age, the LDT scores, the SWPST scores and the Hagerman sentences test scores when Bonferroni correction was used are shown in **Table 2**. Surprisingly, age did not significantly correlate with the LDT scores (*r* = 0.19, *p* > 0.05) but correlated with the SWPST scores (*r* = -0.30, *p* < 0.05) at the verge of significance. However, age significantly correlated with the Hagerman sentences test performance under all six aided conditions (*p* < 0.05, see **Table 2**). This is consistent with previous studies that indicate that there was an age-related decline in speech recognition in adverse listening in older adults compared to younger listeners (Larsby et al., 2008; Gordon-Salant and Cole, 2016). Interestingly, the LDT scores significantly correlated with the Hagerman sentences test performance under 5 out of 6 aided conditions (*p* < 0.05) when NoP, NR, and fast-acting compression (in SSN) were used. The SWPST scores significantly correlated with the Hagerman sentences test performance in 3 out of 6 aided conditions. In addition, the correlation between the LDT scores and the SWPST scores did not significantly correlate (*p* > 0.05), which contrasts with previous studies (Larsby et al., 2005). In summary, verbal information processing speed (LDT scores) was associated with a large benefit from binary masking NR and from fast-acting WDRC (in SSN). WMC (SWPST scores) was related to a larger benefit from binary masking NR but not related to fast-acting WDRC outcomes.

**Table 2:** Correlation matrix of selected predictor variables and speech recognition performance measures (Hagerman test) after applying Bonferroni corrections.

Variables	Age	Lexical decision making (LDT, reaction time, in ms)	Semantic word-pair span test (SWPST)
Age (years)	1		
**Cognitive speed**			
Lexical decision speed test (LDT, reaction time, in ms)	0.19	1	
**Working memory**			
Semantic word-pair span test (SWPST, %)	-30^∗^	-0.14	1
**Hagerman test (dB SNR)**			
Linear amplification, SSN, no noise reduction	0.30*	0.20*	-0.15
Linear amplification, 4TB, no noise reduction	0.30*	0.20*	-0.20*
Linear amplification, SSN, noise reduction	0.27**	0.28*	-0.25*
Linear amplification, 4TB, noise reduction	0.31*	0.23*	-0.20*
Non-linear amplification (Fast-acting compression), SSN, no noise reduction	0.30*	0.30*	-0.14
Non-linear amplification (Fast-acting compression), 4TB, no noise reduction	0.35*	0.15	-0.17

*^∗^*p* < 0.05, ^∗∗^*p* < 0.01.*

In the present study, the focus was on the investigation of how cognitive abilities (i.e., cognitive speed, WMC) may relate to aided speech recognition performance in adverse listening conditions. To perform a more robust test of the prediction concerning the relative involvement of cognitive speed and WMC as predictors of aided speech recognition in noise under six aided conditions, we performed a series of hierarchical multiple regression analyses.

### Hierarchical Multiple Regression Analysis

To investigate the relative role of cognitive speed and WMC in explaining the variance in aided speech recognition in noise performance under six aided conditions, age was controlled to eliminate its confounding effects. After controlling for age, a series of hierarchical regressions was applied under six aided conditions, and in each regression, age was entered in step 1, and the LDT and SWPST were entered in step 2. The results of these hierarchical regressions are shown in **Tables 3, 4**. As shown in **Tables 3, 4**, the LDT scores predicted speech recognition in 5 out of 6 aided conditions after controlling for age, that is, in (1) SSN with NoP, (2) 4TB with NoP, (3) SSN with NR, (4) 4TB with NR, and (5) SSN with fast-acting compression. However, the LDT scores did not predict speech recognition in 4TB with fast-acting compression (*p >* 0.05). The SWPST scores predicted speech recognition performance in only 1 aided condition (*p* < 0.05), beyond age and the LDT. This contrasts with previous studies that found that working memory predicted a large portion of variance when sentences were presented in modulated noise (Gatehouse et al., 2003; Akeroyd, 2008; Rudner et al., 2011; Souza and Arehart, 2015). However, age significantly predicted the decline in speech recognition in all six conditions and explained a large part of the variance when the Hagerman sentences were presented in steady state and 4TB noise background regardless of the hearing aid signal processing algorithms applied.

**Table 3:** Hierarchical regressions predicting speech recognition performance in SSN, 4TB conditions with and without noise reduction processing.

	Step 1			Step 2		
	*b*	*SE b*	β	*b*	*SE b*	β
**In SSN condition without noise reduction**
Constant	-8.67	1.05		-9.93	1.36	
Age	0.076	0.01	0.30^∗∗∗^	0.07	0.02	0.27^∗∗∗^
Lexical decision speed test				0.002	0.001	0.17
Semantic word-pair Span test				-0.02	0.03	
*R*^2^ = 0.10 for step 1, change in *R*^2^ = 0.03 for step 2 (*p* < 0.05)
**In 4TB condition without noise reduction**
Constant	-3.10	1.07		-3.91	1.40	
Age	0.07	0.02	0.30	0.06	0.02	0.25^∗∗∗^
Lexical decision speed test				0.002	0.001	0.17
Semantic word-pair Span test				-0.04	0.03	-0.10
*R*^2^ = 0.09 for step 1, change in *R*^2^ = 0.04 (*p* < 0.05)
**In SSN condition with noise reduction**
Constant	-12.92	1.16		-14.12	1.47	
Age	0.07	0.02	0.27^∗∗∗^	0.06	0.01	0.21
Lexical decision speed test				0.003	0.001	0.24^∗∗∗^
Semantic word-pair Span test				-0.06	0.09	-0.15
*R*^2^ = 0.07 for step 1, change in *R*^2^ = 0.09 (*p* < 0.001)
**In 4TB condition with noise reduction**
Constant	-9.91	1.06		-11.02	1.35	
Age	0.08	0.02	0.31^∗∗∗^	0.07	0.02	0.27^∗∗∗^
Lexical decision speed test				0.002	0.001	0.20
Semantic word-pair Span test				-0.04	0.03	-0.10
*R*^2^ = 0.10 for step 1, change in *R*^2^ = 0.05 (*p* < 0.01)

*b = unstandardized coefficients; SE b = standard errors; *β* = standardized coefficients; ΔR^2^ = change in R^2^^∗^*p* < 0.05, ^∗∗^*p* < 0.01, ^∗∗∗^*p* < 0.001.*

**Table 4:** Hierarchical regressions predicting speech recognition performance in SSN, 4TB conditions, with fast-acting compression.

	Step 1			Step 2		
	*b*	*SE b*	β	*b*	*SE b*	β
**In SSN condition with fast-acting compression**
Constant	-8.51	1–25				
Age	0.09	0.02	30^∗∗∗^	0.08	0.02	0.27^∗∗∗^
Lexical decision speed test				0.004	0.001	0.28^∗∗∗^
Semantic word-pair Span test				-0.01	0.03	-0.03
*R*^2^ = 0.09 for step 1, change in *R*^2^ = 0.08 (*p* < 0.001)
**In 4TB condition with fast-acting compression**
Constant	-3.10	1.07		-3.86	1.41	
Age	0.09	0.02	0.34^∗∗∗^	0.08	0.02	0.32^∗∗∗^
Lexical decision speed test				0.001	0.001	0.12
Semantic word-pair Span test				-0.02	0.03	-0.06
*R*^2^ = 0.12 for step 1, change in *R*^2^ = 0.02 (*p* > 0.05)

*b = unstandardized coefficients; SE b = standard errors; β = standardized coefficients; ΔR^2^ = change in R^2^^∗^*p* < 0.05, ^∗∗^*p* < 0.01, ^∗∗∗^*p* < 0.001.*

## Discussion

The purpose of the present study was to examine the extent to which cognitive abilities (cognitive speed, and WMC) may relate to individual listeners’ responses to digital signal processing settings in adverse listening conditions. We consider adverse listening conditions very generally to mean any background noise (e.g., 4TB) and/or modifications of the acoustic signal (in this study, by noise reduction and fast-acting compression) that may offset the listener’s performance (Larsby et al., 2005; Souza et al., 2015a,b). Overall, our findings are in line with previous studies in showing that cognitive abilities such as WMC and cognitive processing speed play an important role in effective speech recognition in difficult listening environments (Akeroyd, 2008; Rönnberg et al., 2008, 2013, 2016; Rudner et al., 2011; Souza and Arehart, 2015) and decline with age (Salthouse, 1996, 2000). A recent information processing model (ELU; Rönnberg et al., 2013) provides a theoretical background for a better understanding of the relationship observed between cognitive abilities and speech recognition in noise. This model suggests that when the speech signal is presented clearly without distortion, the listener will rapidly and effectively perform a lexical match with the engagement of cognitive resources to a lesser extent (i.e., WMC, processing speed). In contrast, if the speech signal is distorted (caused by either background noise or unwanted signal processing artifacts), then lexical matching may be more difficult, and the listener may have to engage his or her cognitive ability to a greater extent to unlock the meaning of the message or to fill in the missing acoustic information. Our results are consistent with the assumptions of the ELU model.

### The Effects of Hearing Aid Signal Processing and Background Noise

Recent studies have suggested that advances in the hearing aid industry are of great potential benefit to hearing-impaired persons who communicate using the auditory channel (Dillon, 2012; Ng et al., 2013; Souza et al., 2015a,b). In support of this suggestion, several studies have shown relative benefits from fast-acting compression (Gatehouse et al., 2006; Foo et al., 2007; Akeroyd, 2008; Rudner et al., 2011; Souza et al., 2015a,b) and from binary masked noise reduction (Wang et al., 2009; Ng et al., 2013; Rönnberg et al., 2016; Souza et al., 2015b) for persons with hearing impairment. The results of the present study showed that binary masking noise reduction provides a greater benefit than fast-acting compression in adverse listening conditions (Rönnberg et al., 2016). Noise reduction signal processing reduced the adverse effect of modulated noise on speech recognition performance for listeners with good cognitive ability (Souza and Arehart, 2015). This may suggest that the lexical matching of target speech information in long-term memory becomes less explicit and less cognitively taxing (Rönnberg et al., 2008). However, listeners with poor cognitive abilities may not benefit from noise reduction to a greater extent because any significant benefits provided by the signal processing might have been canceled out by the additional cognitive demand exercised by the distortions created by signal processing artifacts (Lunner, 2003; Rudner et al., 2011). On the other hand, fast-acting compression presented limited benefits (relative to the NoP baseline), in support of previous studies that suggested that the fast-acting compression setting may introduce signal distortions or alter the speech envelope, resulting in a phonological mismatch and hence dependence on the listeners’ cognitive capacities (Lunner, 2003; Akeroyd, 2008; Souza and Arehart, 2015). Given that binary masking noise reduction is more aggressive at reducing the effect of background noise, it may have lesser speech signal modification effects than fast-acting compression in terms of speech intelligibility (Wang et al., 2009).

As observed in the two-way interaction, it should be noted that this interaction was driven by the fact that the background noise has a stronger effect in the no processing condition and the fast-acting compression condition compared to noise reduction condition (see the SNR differences; **Figure 2**). This may suggest that background noise rather than cognitive ability is the key factor influencing the interaction effect of signal processing and the noise type on speech recognition performance (relative to the NoP baseline; Larsby et al., 2005). The present findings showed that when noise reduction was applied, speech recognition performance was not significantly different from either that in SSN or in 4TB. That is, when noise reduction was used, the main effect of noise was no longer significant. It may suggest that there was relatively lesser dependence on cognitive resources and more dependence on hearing aid signal processing. On the other hand, speech recognition performance was significantly different in SSN and in 4TB when fast-acting compression was applied (relative to NoP baseline, **Figures 1, 2**). That is, when fast-acting compression was applied, the 4TB disruptive masking effect remained relatively significant, resulting in more dependence on cognitive resources to a greater extent (ELU model, Rönnberg et al., 2008, 2013). The findings also showed that 4TB background noise was more disruptive than SSN, affecting the ease with which a lexical match can be made and taxing cognitive resources (Larsby et al., 2005). Moreover, multiple-talker babble delayed and substantially reduced the benefit obtained from fast-acting compression compared to noise reduction (relative to the NoP baseline; Ng et al., 2013). Our results may also suggest that 4TB may be one of the major contributors to a source of signal degradation in speech recognition performance (see Larsby et al., 2005; Rudner et al., 2011). Moreover, given that the 4TB background noise also consisted of words spoken by two male and two female native Swedish speakers in the present study, the use of native speakers might have stronger masking effects on speech with fast-acting compression than with noise reduction. Our results may suggest that the combination of fast-acting compression and 4TB noise may constitute a major source of degradation that contributes to an impoverishment of the performance on the speech recognition task or to the poor benefit from the hearing instrument. That is, the combination of fast-acting compression-4TB may influence the engagement of explicit cognitive resources to a greater extent than that of noise reduction-SSN does. This finding is in agreement with previous studies (Larsby et al., 2005, 2008; Akeroyd, 2008; Ng et al., 2013). Nevertheless, the combination of binary masking noise reduction and SSN may constitute a minor source of speech signal degradation for hearing-impaired persons (Ng et al., 2013; Rönnberg et al., 2016). We may suggest that older adult hearing aid users may be more vulnerable to the signal degradation created by a combination of the unwanted effects of fast-acting compression and 4TB noise.

### The Effects of Cognitive Speed and Working Memory

The current findings are consistent with the assumption that signal distortion from the combination of fast-acting WDRC and 4TB constitutes a major source of signal degradation that results in an impoverished representation at the auditory periphery. In the context of the information processing models for speech perception, listeners undergoing these multiple sources of signal degradation must assign more processing resources to prior processing phases (Rönnberg et al., 2008; Lunner et al., 2009). This distribution of resources may impoverish the subsequent processes required for the identification of the linguistic content of the sentence materials. Then, a listener may be required to depend on his/her WMC for effective processing of the degraded speech signal and comprehension of the meaning of the message. Nevertheless, when the WMC is limited, this processing may be more difficult or may fail. Our findings show that the WMC scores (SWPST) and cognitive processing speed scores (LDT) did not significantly correlate with speech intelligibility only in 4TB with fast-acting WDRC after using Bonferroni correction and that they did not significantly predict the performance of speech recognition in 4TB with fast-acting compression. In the case of working memory, this finding contrasts with the previously confirmed relationship between working memory and fast-acting compression in a modulated noise background (Gatehouse et al., 2003, 2006; Foo et al., 2007; Rudner et al., 2011). This could be because the amount of resources to be allocated to processing and storage might have been limited or might have exceeded the available capacity, which may result in tax errors, loss of information or slower processing. If we may postulate that stronger fast-acting WDRC processing results in a greater modification of the input signal, then we will expect a relationship between WMC (SWPST scores) and better speech intelligibility (greater benefit) with fast-acting WDRC (Rudner et al., 2011); however, this was not the case in both the correlations and the regression results (see **Tables 2–4**). A possible reason for this could be that stronger fast-acting WDRC processing may be having concurrent effects: improving the audibility of the speech signal while maintaining loudness comfort (by applying gain as a function of intensity, with a lower gain applied to higher input levels) and introducing unwanted processing artifacts that may create more distortions rather than less aggressive processing (Dillon, 2001; Lunner, 2003). Possibly the net effect of these opposing factors contributes to the weak association between the SWPST and fast-acting WDRC (Dillon, 2012).

On the other hand, our results indicate that the effects of a combination of noise reduction and SSN may constitute a minor source of degradation, contributing to a greater benefit or better speech intelligibility. That is, there were significant associations between WMC (SWPST scores) and the binary masking NR outcomes and between the cognitive processing speed (LDT scores) and the binary masking NR outcomes (see **Table 2**). This may be viewed via the ELU model, which assumes a larger influence of cognitive ability (e.g., WMC) when the phonological form of the perceived language signal does not match the phonological representations stored in long-term memory. We may postulate that stronger binary masking NR processing results in a significant modification of the input signal; we would expect a relationship between working memory and better speech intelligibility (or greater benefits from NR processing), and this was the case in the present study. A possible explanation for this could be that stronger binary masking NR processing may have two balanced effects: improving the audibility of the speech signal (as a result of an effective noise suppression), introducing relatively lesser distortion but more aggressive processing (relatively less explicit cognitive resources; Neher, 2014). The results of the present study show that cognitive ability is an important factor in aided speech recognition in adverse listening conditions for persons with hearing impairment.

### Effects of Age

The relationship between age and the measure of cognitive processing speed was observed before applying Bonferroni correction but not after. The pattern of this relationship was contrary to our expectations, in support of previous studies that indicate that processing speed declines with age (Salthouse, 1996; Lunner, 2003; Larsby et al., 2005). However, a relationship between age and the measures of working memory was observed before and after applying Bonferroni correction, suggesting a decline in WMC with age (Salthouse, 1996). As expected, the effects of age on speech recognition in noise performance were observed before and after the use of Bonferroni correction. This pattern of the relationship is in support of previous studies showing poorer performance for older adult listeners compared to younger listeners (Gordon-Salant and Fitzgibbons, 2001; Gordon-Salant and Cole, 2016). The effect of age on speech recognition in noise performance is also underlined by the results of the hierarchical regression analysis, which showed that age, above and beyond the effects of the cognitive processing speed and working memory, contributed to variance in the intelligibility scores, suggesting that hearing aid users may have age-related degradations in higher-level processing that extend beyond what is captured in the SWPST and the SDT. This may suggest that there are other factors associated with aging other than cognitive functioning that may be involved in the age-related decline in speech recognition among older hearing aid users (Baltes and Lindenberger, 1997; Wingfield and Tun, 2001; Larsby et al., 2008).

## Conclusion

The present study adds to previous studies a new way of viewing the relationship between cognitive abilities and binary masking noise reduction and fast-acting WDC for speech recognition in noise for hearing aid users (Lunner, 2003; Akeroyd, 2008; Ng et al., 2013; Souza and Arehart, 2015). The results showed that after controlling for age, cognitive processing speed was a better predictor of speech intelligibility in noise (in both SSN and 4TB), suggesting a significant association between cognitive processing speed (measured by LDT) and binary masking NR and fast-acting compression (in SSN). However, there were weaker associations between WMC (measured by the SWPST) and speech intelligibility in noise with NR, and no association when fast-acting WDRC was used. That is, WMC was a weaker predictor of speech intelligibility in noise. The findings might have been different if the participants had been provided with training and/or allowed to acclimatize to binary masking noise reduction or fast-acting compression (Rudner et al., 2011). Taken together, the results suggest that assessing the effects of cognitive processing speed, WMC, and hearing aid signal processing settings in noise may provide important insights into the source of hearing aid users’ complaints of difficulty recognizing speech in noise.

## Ethics Statement

The subjects were informed about the voluntariness, confidentiality and they were free to decline participation at any time. They also filled up a written informed consent form in which they give the researchers the right to collect and restore personal information relevant to the study. Personal information was collected and stored in a way that participant’s integrity was maximized (Breakwell et al., 2006).

## Author Contributions

WY has substantially contributed to the following phases of the present study: the conception, the design, the acquisition, analysis, and the interpretation of data; the draft and critical revision; the final approval of the version to be published. The author is accountable for all aspects of this work.

## Conflict of Interest Statement

The author declares that the research was conducted in the absence of any commercial or financial relationships that could be construed as a potential conflict of interest.
